# Lemon Peel Water Extract: A Novel Material for Retinal Health, Protecting Retinal Pigment Epithelial Cells against Dynamin-Related Protein 1-Mediated Mitochondrial Fission by Blocking ROS-Stimulated Mitogen-Activated Protein Kinase/Extracellular Signal-Regulated Kinase Pathway

**DOI:** 10.3390/antiox13050538

**Published:** 2024-04-27

**Authors:** Shang-Chun Tsou, Chen-Ju Chuang, Inga Wang, Tzu-Chun Chen, Jui-Hsuan Yeh, Chin-Lin Hsu, Yu-Chien Hung, Ming-Chung Lee, Yuan-Yen Chang, Hui-Wen Lin

**Affiliations:** 1 Department of Nutrition, Chung Shan Medical University, Taichung 40201, Taiwan; 0946003@live.csmu.edu.tw (S.-C.T.); clhsu@csmu.edu.tw (C.-L.H.); 2 Emergency Department, St. Martin De Porres Hospital, Chiayi 60069, Taiwan; ilovespurs168@gmail.com; 3 Rehabilitation Sciences & Technology, University of Wisconsin-Milwaukee, Milwaukee, WI 53211, USA; wang52@uwm.edu; 4 Institute of Medicine, Chung Shan Medical University, Taichung 40201, Taiwan; 1103012@live.csmu.edu.tw (T.-C.C.); 1003023@live.csmu.edu.tw (J.-H.Y.); 5 Department of Ophthalmology, Chung Shan Medical University Hospital, Taichung 40201, Taiwan; b92401086@ntu.edu.tw; 6 Brion Research Institute of Taiwan, New Taipei City 23143, Taiwan; i1es@herbbiotek.com; 7 Department of Microbiology and Immunology, School of Medicine, Chung Shan Medical University, Taichung 40201, Taiwan; 8 Clinical Laboratory, Chung Shan Medical University Hospital, Taichung 40201, Taiwan; 9 Department of Optometry, Asia University, Taichung 413305, Taiwan

**Keywords:** sodium iodate (NaIO_3_), lemon peel ultrasonic-assisted water extract (LUWE), retinal degeneration, reactive oxygen species (ROS), apoptosis

## Abstract

Previous studies showed that NaIO_3_ can induce oxidative stress-mediated retinal pigment epithelium (RPE) damage to simulate age-related macular degeneration (AMD). Lemon peel is rich in antioxidants and components that can penetrate the blood–retinal barrier, but their role in retinal oxidative damage remains unexplored. Here, we explore the protection of lemon peel ultrasonic-assisted water extract (LUWE), containing large amounts of flavonoids and polyphenols, against NaIO_3_-induced retinal degeneration. We initially demonstrated that LUWE, orally administered, prevented retinal distortion and thinning on the inner and outer nuclei layers, downregulating cleaved caspase-3 protein expression in RPE cells in NaIO_3_-induced mice. The effect of LUWE was achieved through the suppression of apoptosis and the associated proteins, such as cleaved PARP and cleaved caspase-3, as suggested by NaIO_3_-induced ARPE-19 cell models. This is because LUWE reduced reactive oxygen species-mediated mitochondrial fission via regulating p-Drp-1 and Fis1 expression. We further confirmed that LUWE suppresses the expression of p-MEK-1/2 and p-ERK-1/2 in NaIO_3_-induced ARPE-19 cells, thereby providing the protection described above, which was confirmed using PD98059 and U0126. These results indicated that LUWE prevents mitochondrial oxidative stress-mediated RPE damage via the MEK/ERK pathway. Elucidation of the molecular mechanism may provide a new protective strategy against retinal degeneration.

## 1. Introduction

Age-related macular degeneration (AMD), the third leading cause of blindness [1], is characterized by the atrophy of photoreceptors, caused by dysfunction of the retinal pigment epithelium (RPE), which is responsible for retinal physiology and toxicology [2]. Reactive oxygen species (ROS) accumulation with age is the primary cause of RPE cell senescence and apoptosis [3], triggering lysosomal acidification, DNA fragmentation, and mitochondrial fission [4,5]. Sodium iodate (NaIO_3_), a natural oxidizing agent, has become an effective model for simulating AMD disease due to its specificity to induce the symptoms described above [6]; however, its complexity in mitochondrial homeostasis requires further clarification [7,8]. The mitochondrial fission regulators, dynamin-related protein 1 (Drp1) and mitochondrial fission protein 1 (Fis1), stabilize mitochondrial function by regulating mitochondrial dynamics. However, they can also promote intrinsic apoptosis by accelerating cytochrome c into the cytoplasm through Bcl2-associated X protein (Bax) [9,10].

MEK/ERK (mitogen-activated protein kinase/extracellular signal-regulated kinase) signaling may be a risk factor for RPE mitochondrial dysfunction [11]. ERK is traditionally recognized for cell proliferation and survival; however, it can stimulate pro-apoptotic signals, initiate Ca^2+^-dependent endoplasmic reticulum stress, and induce retinal pigment epithelial–mesenchymal transition (EMT) under ROS stimulation [12]. Furthermore, it was revealed that NaIO_3_ triggers Drp-1-mediated mitochondrial fission to initiate cell death through the MAPK pathway [4]. Accumulating evidence, in conjunction with the above concepts, suggests that mitochondrial dynamics play a crucial role in NaIO_3_-induced cell death.

While anti-vascular endothelial growth factor (VEGF) agents and laser therapy have been employed in treating choroidal neovascularization, the FDA (U.S. Food and Drug Administration) has not approved any treatment strategies for retinal atrophy [13]. The bioavailability of most antioxidant formulas is limited by the blood–retinal barrier (BRB) [14]. Therefore, research into new materials may offer a promising avenue for AMD.

Lemon (*Citrus limon*) is widely popular and consumed for its unique flavor. However, as it is primarily used for juicing, over 50% of the total fruit weight (mainly consisting of peels and seeds) often goes underutilized, leading to environmental pollution or soil acidification [15]. The peel of lemon is a rich source of polyphenols and flavonoids [16,17], such as hesperidin and naringin, possessing strong antioxidant properties and blood–brain barrier permeability [18,19]. Quercetin, a citrus flavonone, can also protect RPE cells against oxidative damage [20]. In addition, recent research has emphasized the synergistic potential of natural formulations in terms of bioavailability [21,22], while the high content of vitamin C in lemon has been reported to enhance the bioavailability of lutein in rats [23]. These findings engender our expectations of the potential benefits of lemon by-products in addressing AMD.

Here, we evaluated the protection of lemon peel water extract against retinal degeneration using NaIO_3_-induced ARPE-19 cells (a human RPE cell line) and a mouse model. We further investigated its mechanism for reducing p-Drp-1-mediated mitochondrial fission and RPE cell apoptosis, by inhibiting the MEK/ERK pathway. This study first reports the protective effect of lemon peel extract against retinal oxidative damage and provides a novel protective strategy.

## 2. Material and Methods

### 2.1. Chemicals

The 2,2′-azino-bis (3-ethylbenzothiazoline-6-sulfonic acid) (ABTS, CAS 30931-67-0), hydrogen peroxide (H_2_O_2,_ CAS 7722-84-1), peroxidase (CAS 9003-99-0), 2,2-diphenyl-1-picrylhydrazyl (DPPH, CAS 1898-66-4), sodium nitrite (NaNO_2,_ CAS 7632-00-0), aluminum chloride (AlCl_3_, CAS 7446-70-0), sodium hydroxide (NaOH, CAS 1310-73-2), Folin–Ciocalteu’s phenol reagent (CAS F9252), and sodium carbonate (Na_2_CO_3_, CAS 56169) were all purchased from Merck (Rahway, NJ, USA). U0126 (Cat. HY-12031A) and PD98059 (Cat. HY-12028) were purchased from MedChemExpress (Monmouth Junction, NJ, USA).

### 2.2. Preparation of LUWE

This study utilized Eureka lemon (*Citrus limon*) sourced from Pingtung, Taiwan. The by-products of lemon peel, including the endocarp residual membranes and exocarp, are the residues generated after juicing. The samples were washed, seeds removed, and diced to 0.5–1 cm^3^. Then, the samples were soaked in 95 °C distilled water at a ratio of 1:10 (g/mL) for 1 min for sterilization, referring to the methodology of Karbuz and Tugrul [24]. After that, the lemon peel was cooled down in an ice-cold water bath, followed by the extraction.

The ultrasonic-assisted water extraction (UWE) procedure was conducted with reference to previous literature, incorporating some modifications [25,26]. Extraction solutions were prepared at a peel/solvent ratio of 1:10 (g/mL) as described above. Extraction was performed using a 2 L ultrasonic bath with a frequency of 43 ± 2 kHz and a power of 80 W (Homelike Electronic CO., LTD., Kaohsiung, Taiwan) for 1 h at 50 °C. The extracts were filtered through double-layer gauze (Cat. 161363, Good Verita Enterprise CO., LTD., Taipei, Taiwan), and concentrated by freeze-drying (BTP-9ES, Benchtop Pro, Wolflabs, Pocklington, UK) for experimental use.

### 2.3. Trolox Equivalent Antioxidant Capacity Assay (TEAC)

The rapid ABTS^+^ peroxidase assay protocol was adapted from Konan et al. [27]. In brief, the ABTS^+^ working solution was prepared by mixing equal volumes of 100 µM ABTS, 4.4 units/mL peroxidase, and 50 µM H_2_O_2_. Subsequently, 30 μL of the sample was added to 970 μL of the ABTS^+^ working solution, in the dark at room temperature for 1 min. A 200 μL aliquot of the mixture was transferred to a 96-well plate, and the absorbance was measured at 410 nm using an ELISA reader to establish a standard curve and calculate the half maximal inhibitory concentration (IC50). Trolox was used as a standard, and the interpolation method was employed to calculate the sample’s Trolox equivalent antioxidant activity.

### 2.4. DPPH Radical Scavenging Activity

The DPPH free radical scavenging activity experiment, as described by Anggraini et al., was conducted [28]. A 1 mM DPPH working solution was prepared by dissolving DPPH in methanol and mixing it thoroughly. Then, 200 µL of the sample and 50 µL of the DPPH working solution were added to a 96-well plate and allowed to react in the dark at room temperature for 30 min. The absorbance was measured at 517 nm using an ELISA reader to establish a standard curve and calculate the half maximal inhibitory concentration (IC50). Finally, the catechin equivalent antioxidant activity was calculated using interpolation compared to the standard curve of catechin.

### 2.5. Analysis of Flavonoid Content

The total flavonoid content of the samples was determined using the aluminum chloride method as reported by Shraim et al. [29]. Firstly, 250 µL of the sample was mixed with 75 µL of 5% NaNO_2_ and reacted for 6 min. Then, 150 µL of 10% AlCl_3_ was added and reacted for 5 min. Finally, 0.5 mL of 1M NaOH was added, and the absorbance was measured at 510 nm using an ELISA reader. A standard curve was established, and the content of flavonoids in terms of their catechin equivalent was calculated using the interpolation method and compared with the catechin standard curve.

### 2.6. Analysis of Polyphenol Content

The total polyphenol content of the samples was determined following the method described by Carmona-Hernandez et al. [30]. The amount of 50 µL of the sample was mixed with 0.5 mL of 1N Folin–Ciocalteu’s phenol reagent and 2.5 mL of 10% (*w*/*w*) Na_2_CO_3_. The mixture was allowed to react in the dark at room temperature for 20 min. Next, 200 µL of the mixture was transferred to a 96-well plate, and the absorbance was measured at 700 nm using an ELISA reader. A standard curve was constructed and compared with the gallic acid standard curve using the interpolation method to calculate the gallic acid equivalent polyphenol content.

### 2.7. Analysis of LUWE Components

The components of LUWE were analyzed using 3D high-performance liquid chromatography (3D-HPLC) with a photodiode array detector (Burdick & Jackson, Seongnam, Republic of Korea) to generate 3D graphs, and identification was carried out through liquid chromatography/mass spectrometry (LC/MS).

The HPLC column dimensions are 4.6 mm × 250 mm × 5 µm, with the mobile phase consisting of solvent A (0.1% TFA aqueous solution) and solvent B (0.1% TFA acetonitrile solution). HPLC parameters include a column temperature of 35 °C, a flow rate of 1.0 mL/min, and an analysis time of 65 min.

The LC/MS system comprises the Shimadzu LC-20AD UFLC and LCMS-8040 mass spectrometer. The mobile phase consisted of solvent A (0.1% formic acid and 1 g/L ammonium acetate in distilled water) and solvent B (0.1% formic acid and 1 g/L ammonium acetate in distilled water), with methanol used for gradient elution. The gradient curve was as follows: 0–40 min, A decreases from 100% to 70%, and B increases from 0% to 30%; 40–70 min, A decreases from 70% to 0%, and B increases from 30% to 100%. At 70–70.1 min, A is held at 0%, and B becomes 100%. From 70.1 to 80 min, A is maintained at 100%, and B at 0%. The flow rate is 0.4 mL/min, the column temperature is 40 °C, the injection volume is 30 μL, and a Shim-pack XR-ODS II column is used.

MS was conducted using electrospray ionization (ESI) in dual ionization modes ([ESI][+] and [−]), with a full scan range of 400–800 amu. The interface voltage for ESI (+) was set at 4.5 kV, and for ESI (−), it was −3.5 kV. Nitrogen was used for nebulization and drying, with flow rates of 3.0 and 10 L/min, respectively. Collision-induced dissociation gas was argon, maintained at 230 kPa. The desolvation line temperature was set to 150 °C, and the heat block temperature was maintained at 400 °C.

### 2.8. Animal Model

Eight-week-old Balb/c mice from the National Laboratory Animal Center of Taiwan (Taipei, Taiwan) were housed in compliant cages with a 12/12 h light/dark cycle and free access to food and water. Under the guidance of the Institutional Animal Care and Use Committee of Chung Shan Medical University (IACUC number: 2595), mice were randomly divided into three groups (*n* = 6) for the following treatments:

NaIO_3_ + LUWE: Mice received a daily oral feed of 200 µL LUWE (100 mg/ mL in PBS) for 7 days, followed by an intravenous (IV) injection of NaIO_3_ (40 mg/ kg in PBS).

NaIO_3_: Mice received a daily oral feed of 200 µL PBS for 7 days, followed by an intravenous injection of NaIO_3_ (40 mg/ kg).

Mock: Mice received a daily oral feed of 200 µL PBS) for 7 days, followed by an intravenous injection of 100 µL PBS.

All mice were continuously fed as described after receiving intravenous injections (only once) until sacrifice.

### 2.9. Tissue Sections and Thickness Measurement

All mice were sacrificed on the 7th day following the intravenous injection of NaIO_3_. Their right eyes were collected and fixed with 10% formalin. The fixed eye tissues were sectioned and stained with hematoxylin/eosin. As described in previous studies [20,31], the thickness of the ONL (outer nuclear layer) and INL (inner nuclear layer) of the retina was measured at 50 μm intervals within 600 μm to 900 μm outward from the center of the retina or optic nerve, and the average was calculated. The immunohistochemical staining of cleaved caspase-3 (31A106, Santa Cruz) was performed using the BondMax automated staining system (Vision BioSystems Ltd., Newcastle Upon Tyne, UK).

### 2.10. Human RPE Cell Model

The ARPE-19 cell line (adult male RPE cells) was purchased from the American Type Culture Collection (Manassas, VA, USA). Cells were cultured in DMEM (Dulbecco’s Modified Eagle’s Medium, Hyclone, Grand Island, NY, USA) containing 10% FBS (Gibco, Grand Island, NY, USA) with 100 U/mL penicillin and streptomycin (Gibco, Grand Island, NY, USA) at 37 °C and 5% CO_2_. The ARPE-19 cells were seeded into a 12-well plate at a density of 2.0 × 10^5^ cells per well and incubated for 12 h to ensure cell recovery and adhesion before conducting the experiments.

### 2.11. Cell Viability Assay

After processing according to the design, ARPE-19 cells were treated with 2% CCK-8 (Dojindo Molecular Technologies, Tokyo, Japan) and incubated at 37 °C for 1 h. The absorbance of each group was quantified by an ELISA reader at a wavelength of 450–595 nm and adjusted according to the color caused by the acidity of the sample.

### 2.12. Cell Morphology and Apoptosis Analysis

Following the designated treatments, morphological changes in ARPE-19 cells from each group were recorded, and subsequently, cell separation was performed using 0.25% Trypsin–EDTA (Gibco, Grand Island, NY, USA). The separated ARPE-19 cells were stained with annexin V-FITC (0.25 µg/mL) and propidium iodide (1 µg/mL) for 1 h, and the fluorescence was detected by flow cytometry (BD Biosciences, San Jose, CA, USA). FITC is detected at 515 nm, and PI is detected at 590 nm.

### 2.13. Mitochondrial ROS Level Analysis

The ARPE-19 cells in each group were replaced with DMEM containing 4 µM MitoSOX Red (M36008, Thermo Fisher Scientific, Waltham, MA, USA) for 30 min. The cells were washed twice with 1X PBS and then detached with 0.25% Trypsin–EDTA from a 12-well plate. The fluorescence content was detected using a flow cytometer (Accuri C6 Plus, BD Bioscience, San Jose, CA, USA) with an excitation wavelength of 590 nm.

### 2.14. Western Blotting

ARPE-19 cells in each group were removed from the supernatant and lysed with cell lysis buffer (containing 10 mM Tris, 0.1% Triton X-100, and 1 mM EDTA) to collect protein samples. To analyze the expression levels of specific proteins, all samples were separated by 10% SDS-PAGE gel electrophoresis and transferred to PVDF membranes for binding with corresponding antibodies. After adding ECL substrate, all proteins were visualized using MultiGel-21 (GIS-21-C2-6M, TOPBIO) for imaging (Taipei, Taiwan). The antibodies, including cleaved caspase-3 (31A106), p-MEK-1/2 (7E10), p-ERK (12D4), p-p38 (D8), p-JNK (G7), and Fis1 (B5) were all purchased from Santa Cruz Biotechnology (Santa Cruz, CA, USA). Cleaved caspase-9 (9505T), cleaved PARP (Asp214, D64E10), and p-Drp-1 (3455S) were purchased from Cell Signaling Technology (Beverly, MA, USA).

### 2.15. Mitochondrial Dimension Measurements

We inoculated 1 × 10^6^ cells into a 12-well plate. Following experimental treatments as per the design, we washed the cells twice with 1 X PBS, then separated them using 0.25% Trypsin–EDTA. Subsequently, mitochondria were isolated using a Mitochondria Isolation Kit (Catalog number 89874, Thermo Fisher Scientific, Waltham, MA, USA) following the manufacturer’s instructions. The size of mitochondrial particles was measured using forward scatter light (FSC-A) on a flow cytometer (NovoCyte, Agilent Technologies, Santa Clara, CA, USA), following the methods reported by Tzur et al. and Stern et al. [32,33].

### 2.16. Statistical Analysis

All data were analyzed using SAS version 9.4 (SAS Institute Inc., Cary, NC, USA), and all values were expressed as mean ± standard deviation (SD). One-way analysis of variance (ANOVA) was used to determine differences in all data from in vitro and in vivo experiments, and Tukey’s HSD test was used to further analyze significant differences between groups. The significance level was set at 0.05.

## 3. Results

### 3.1. Analysis of Antioxidant Activity in LUWE

We prepared a lemon ultrasonic-assisted water extract (LUWE), referring to the previous literature. This method has been proven effective in separating antioxidant components and maintaining their activity [25,26]. LUWE samples were analyzed for antioxidant activity within 6 h after extraction and were stored at 4 °C during this period. In Table 1, the LUWE exhibited an antioxidant capacity in ABTS^+^ (5.65 ± 0.06 mmole Trolox/g LUWE) and DPPH (2.51 ± 0.03 mmole catechin/g LUWE).

**Figure 1:**
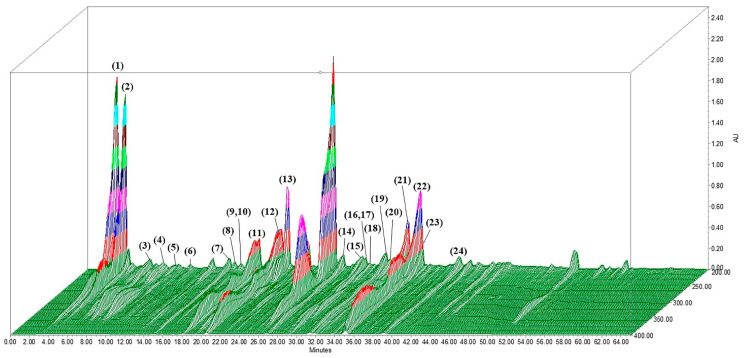
The 3D-HPLC chromatographic fingerprint analysis of LUWE. The chemical compositions of the LUWE samples were analyzed using high-performance liquid chromatography (HPLC) with a photodiode array detector (PDA). Twenty-four polyphenolic or flavonoid compounds were detected and are displayed in Table 2 according to their corresponding peak numbers. AU = arbitrary perfusion units.

We determined the content of polyphenols and flavonoids in these samples using the Folin–Ciocalteu reagent and aluminum chloride, as these are typically the sources of antioxidant activity in natural extracts [34]. LUWE contains a 10.24 ± 0.09 mmole gallic acid/g LUWE polyphenol content and a 964 ± 46.61 µmoles catechin/g LUWE flavonoid content. Based on the previous literature, we established a chromatogram using 3D-HPLC (Figure 1) and identified polyphenols and flavonoids in LUWE through LC/MS analysis (Table 2). The chromatogram illustrates 24 polyphenols and flavonoids present in LUWE. Specifically, LUWE exhibited high concentrations of isocitric acid, citric acid, limocitrin-Glu-HMG-Glu, neoeriocitrin, p-coumatric acid, luteolin, and limocitrin.

### 3.2. LUWE Mitigates Retinal Degeneration in Morphology and Thickness Induced by NaIO_3_

**Figure 2:**
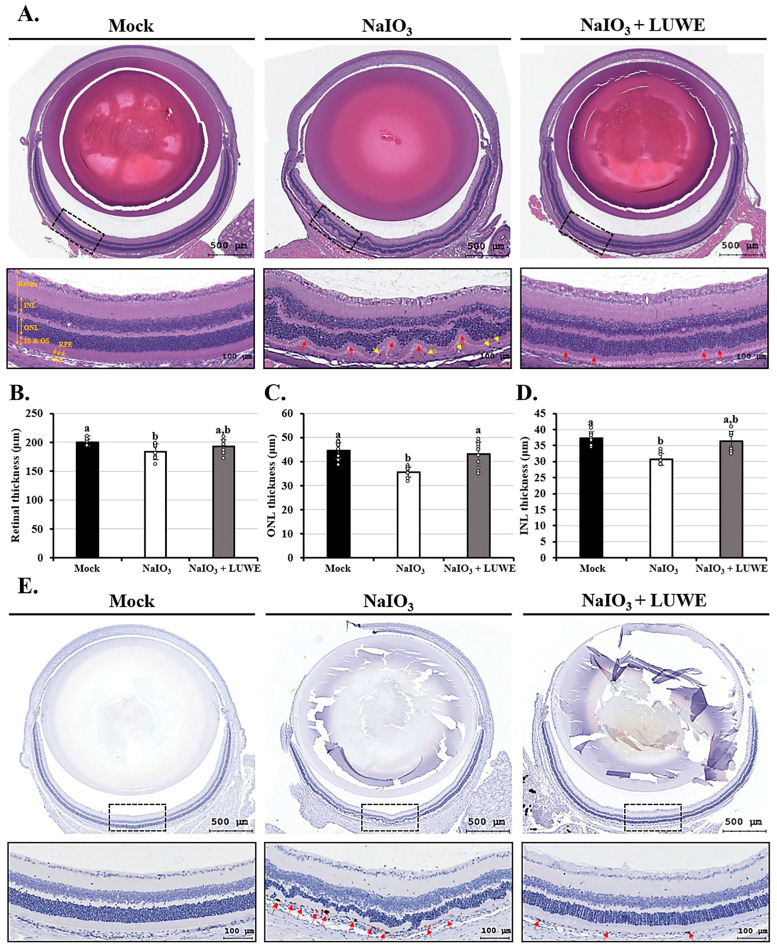
Effects of LUWE on retinal histological changes in Balb/c mice induced by NaIO_3_. All mice were sacrificed after 7 days of intravenous injection. Mouse eyeballs were (**A**) stained with hematoxylin and eosin to quantify the thickness of the (**B**) retina, (**C**) ONL, (**D**) INL, or (**E**) immunohistochemically stained with cleaved caspase-3 antibody. Scale bar = 100 µm. The red arrows marked irregular deformations at the base of the retina, while the yellow arrows indicated migrated cell nucleus. All data on thickness were expressed as mean ± standard deviation (*n* = 6). Different letters (a,b) superscripted in the statistical chart indicate that there is statistical significance between groups (*p* < 0.05); on the contrary, being marked with the same letter indicates that there is no statistical significance.

To confirm retinal protection, mice were orally administered 100 mg/mL LUWE for 7 days, followed by a tail vein injection of 40 mg/kg NaIO_3_ [20,31]. Our results showed that intravenous injection of NaIO_3_ resulted in numerous obvious elevations on the 7th day, originating from the basal layer of the retina (indicated by red arrows). Additionally, NaIO_3_ disrupted the boundaries between the inner nuclear layer (INL) and outer nuclear layer (ONL) and led to unidentified nuclei migration (indicated by yellow arrows), observed in the inner segments (ISs) and outer segments (OSs) (Figure 2A). These nuclei may originate from RPE cells, photoreceptor cells, or choroidal endothelial cells [45,46]. However, LUWE significantly relieved the retinal morphological changes induced by NaIO_3_, resulting in clearer boundaries between each layer (Figure 2A). These results demonstrate that LUWE can protect the structural integrity of the retina against the pathological features induced by NaIO_3_.

We further measured the average thickness of the INL and ONL based on H&E staining results. The total retinal thickness of the mock group was 199.57 ± 6.1 μm (Figure 2B), with an average thickness of 44.65 ± 3.29 μm for the ONL (Figure 2C) and 37.22 ± 1.99 μm for the INL (Figure 2D). NaIO_3_ significantly reduced the average thickness of both the ONL (35.60 ± 2.16 μm) and INL (30.72 ± 1.78 μm), consequently decreasing the total retinal thickness (83.74 ± 13.59 μm). In contrast, LUWE maintained the thickness of the ONL (43.24 ± 4.99 μm) and INL (35.60 ± 2.16 μm), thereby protecting the total retinal thickness (43.24 ± 4.99 μm).

The apoptosis regulated by the caspase cascade following NaIO_3_ exposure has been previously confirmed as a major cause of retinal oxidative damage [7]. Our immunohistochemical staining showed that NaIO_3_ only promoted the expression of cleaved caspase-3 protein in RPE cells (marked by red arrows), while other cells did not express it. In LUWE-pretreated retina, the above-mentioned protein expression was significantly diminished (Figure 2E). Past reports indicate that the INL and ONL rely on intact RPE cells to maintain their physiological metabolism and assist in the removal of RPE toxicity [8], suggesting that LUWE may prevent NaIO_3_-induced retinal degeneration by inhibiting RPE cell apoptosis. Therefore, the molecular mechanisms underlying this protection were further investigated.

### 3.3. LUWE Attenuates Cell Death in NaIO_3_-Treated ARPE-19 Cells

**Figure 3:**
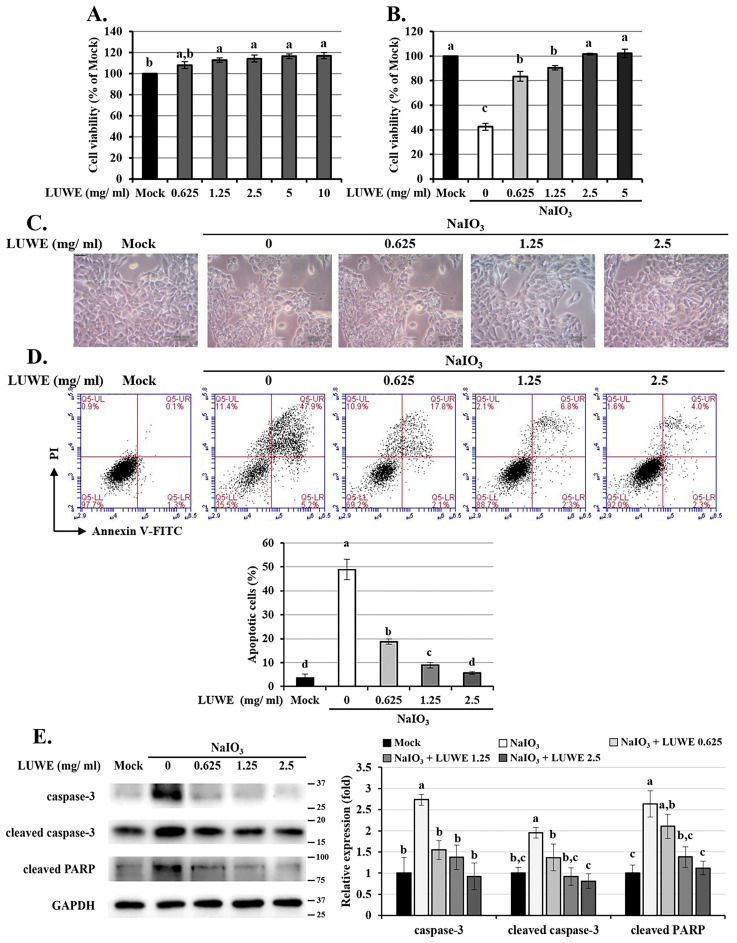
Protection of LUWE in NaIO_3_-induced ARPE-19 cell apoptosis. ARPE-19 cells were (**A**) treated with different doses of LUWE, or (**B**) pretreated with LUWE as designed for 1.5 h, and then co-cultured with 6 mM NaIO_3_ for 24 h to detect cell viability. (**C**) The cell morphology was recorded (bar = 10 µm), and (**D**) annexin V/PI staining was performed. The percentage of apoptotic cells is the sum of early apoptotic cells (Q5-LR: annexin V+/PI−) and late apoptotic cells (Q5-UR: annexin V+/PI+). (**E**) Cell lysates were collected for analysis of caspase-3, cleaved caspase-3, and cleaved PARP protein expression by Western blotting. Protein expression is represented by the fold of the mock group. All data are expressed as the mean ± SD (*n* = 3). Different letters (a–d) superscripted in the statistical chart indicate that there is statistical significance between groups (*p* < 0.05); on the contrary, being marked with the same letter indicates that there is no statistical significance.

To confirm the effect of LUWE on RPE cells, we pretreated ARPE-19 cells (an adult RPE cell line) with different concentrations of LUWE for 1.5 h, followed by co-incubation with 6 mM NaIO_3_ for 24 h, which induced approximately 50% cell death, as previously reported by our group [20,31]. The results showed that 10 mg/mL LUWE slightly increased the viability of ARPE-19 cells (Figure 3A), and a concentration as low as 2.5 mg/mL effectively prevented NaIO_3_-induced cell death (Figure 3B).

We observed that NaIO_3_ stimulated morphological changes in ARPE-19 cells characterized by cell swelling and vacuolation, reminiscent of the apoptotic cells described by Godwin et al. [47] (Figure 3C). Therefore, annexin V/PI staining was performed to determine the pathway of cell death according to the method described by Balmer et al. [7]. The results showed that NaIO_3_ induced 48.9 ± 4.2% apoptotic cells, while 0.625, 1.25, and 2.5 mg/mL of LUWE reduced the apoptotic cells to 18.75 ± 1.15%, 7.9 ± 1.2%, and 5.65 ± 0.65%, respectively (Figure 3D).

We also collected cell lysates from each group for further analysis of the expression of the pro-apoptotic proteins caspase-3 and PARP [7]. As shown in Figure 3E, LUWE not only inhibited the activated forms of caspase-3 and PARP but also reduced the full-length expression of these proteins. These results confirm that LUWE can protect ARPE-19 cell viability by suppressing the apoptotic pathway induced by NaIO_3_.

### 3.4. LUWE Attenuates the Mitochondrial ROS-Stimulated MEK/ERK Signaling Pathway in NaIO_3_-Treated ARPE-19 Cells

**Figure 4:**
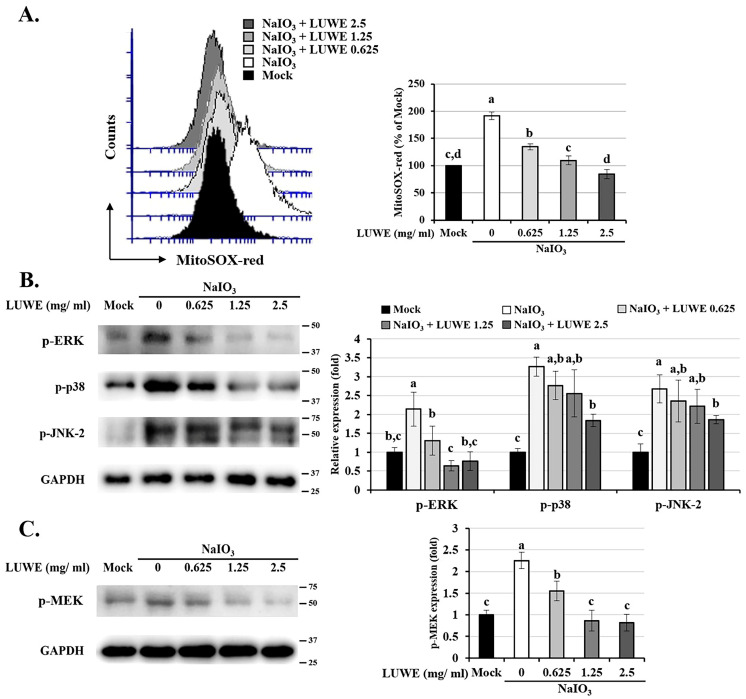
Effects of LUWE on the mitochondrial ROS level and MAPK proteins induced by NaIO_3_. After treatment according to the experimental design, ARPE-19 cells in each group (**A**) were analyzed for mitochondrial ROS levels using MitoSOX Red staining or were collected for analyzing (**B**) the p-ERK, p-p38, p-JNK2, and (**C**) p-MEK protein expression by Western blotting. MitoSOX Red in each group was quantified as a percentage compared with the fluorescent values of the mock group. Protein expression was represented by the fold of the mock group. All data are expressed as the mean ± SD (*n* = 3). Different letters (a–d) superscripted in the statistical chart indicate that there is statistical significance between groups (*p* < 0.05); on the contrary, being marked with the same letter indicates that there is no statistical significance.

Mitochondrial oxidative stress is one of the major factors accelerating the progression of AMD by driving RPE cell autophagy, DNA damage, or cell cycle arrest, resulting in cellular senescence and apoptosis [48]. Our MitoSOX Red staining demonstrated that LUWE could dose-dependently inhibit mitochondrial ROS production in NaIO_3_-treated ARPE-19 cells (Figure 4A). Therefore, we further collected the cell lysates from each group to analyze the expression of mitogen-activated protein kinases (MAPKs), including p-ERK, p-p38, and p-JNK. As crucial secondary messengers in cells, this pathway directly mediates RPE cell mesenchymal transition, autophagy, and apoptosis under oxidative stress or hypoxic conditions [12,49]. In NaIO_3_-induced ARPE-19 cells, LUWE inhibited the expression of the above-mentioned proteins, with the most significant difference observed in p-ERK (Figure 4B). Therefore, we also analyzed the expression of its core upstream factor, p-MEK-1/2 [50], and confirmed its consistent trend with p-ERK across all cell groups (Figure 4C). These results suggest that LUWE may primarily target ROS-regulated MEK/ERK signaling, thereby protecting against NaIO_3_-induced cell death in ARPE-19 cells.

### 3.5. LUWE Inhibits the Intrinsic Apoptosis Induced by NaIO_3_ through the MEK/ERK Signaling Pathway

**Figure 5:**
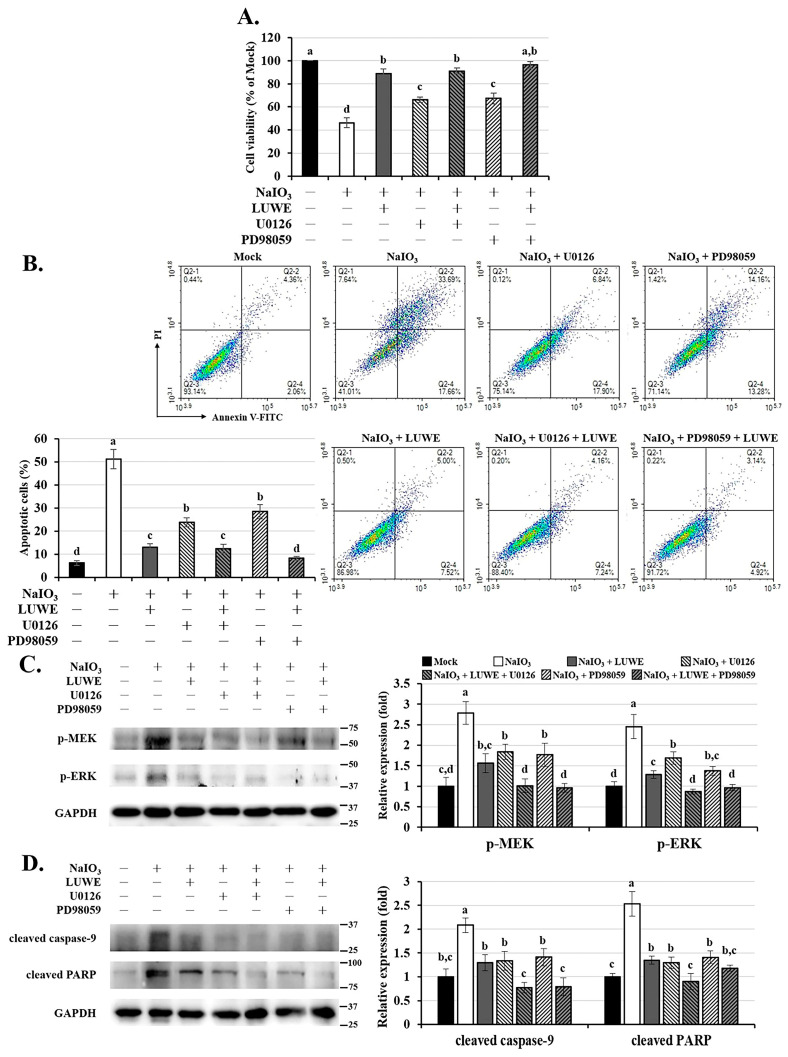
Effects of LUWE, U0126, and PD98059 on NaIO_3_-induced ARPE-19 cell apoptosis. ARPE-19 cells were pretreated with 10 µM U0126 or 20 µM PD98059 mixed with 2.5 mg/ mL LUWE or not for 1.5 h, and then cotreated with NaIO_3_ for 24 h. (**A**) The cell viability was analyzed using the CCK-8 reagent, and (**B**) the ratio of apoptotic cells was determined by annexin V/PI staining. Cell lysates were collected for analysis of (**C**) p-MEK-1/2, p-ERK-1/2, (**D**) cleaved caspase-9 PARP, and cleaved PARP protein expression by Western blotting. Protein expression was represented by the fold of the mock group. All data are expressed as the mean ± SD (*n* = 3). Different letters (a–d) superscripted in the statistical chart indicate that there is statistical significance between groups (*p* < 0.05); on the contrary, being marked with the same letter indicates that there is no statistical significance.

To confirm the role of MEK/ERK signaling in NaIO_3_-induced cytotoxicity, we pretreated ARPE-19 cells with 20 µM MEK inhibitor PD98059 or 10 µM ERK inhibitor U0126 before co-culturing them with NaIO_3_ [51]. In Figure 5A, it can be seen that U0126 and PD98059 can significantly restore the cell viability induced by NaIO_3_. Meanwhile, the results of annexin V/PI staining showed that U0126 and PD98059 effectively reduced apoptotic ARPE-19 cells (Figure 5B). The above effects were further enhanced upon cotreatment with LUWE.

Therefore, we also examined the expression of pro-apoptotic proteins using Western blotting. We first confirmed that U0126 and PD98059 inhibited the expression of p-MEK-1/2 and p-ERK-1/2 induced by NaIO_3_, which showed a positive correlation with cytotoxicity (Figure 5C). Furthermore, we observed that U0126, PD98059, and LUWE could inhibit NaIO_3_-induced cleaved PARP protein expression, consistent with the trend of cleaved caspase-9 (Figure 5D), which is the initiator of intrinsic apoptotic bodies triggered by mitochondrial damage [52]. Based on the above results, we understand that LUWE protects against intrinsic apoptosis in RPE cells by reducing mitochondrial oxidative stress-stimulated MEK/ERK pathways.

### 3.6. LUWE Reduces Mitochondrial Fission Regulated by the MEK/ERK Signaling Pathway in NaIO_3_-Treated ARPE-19 Cells

**Figure 6:**
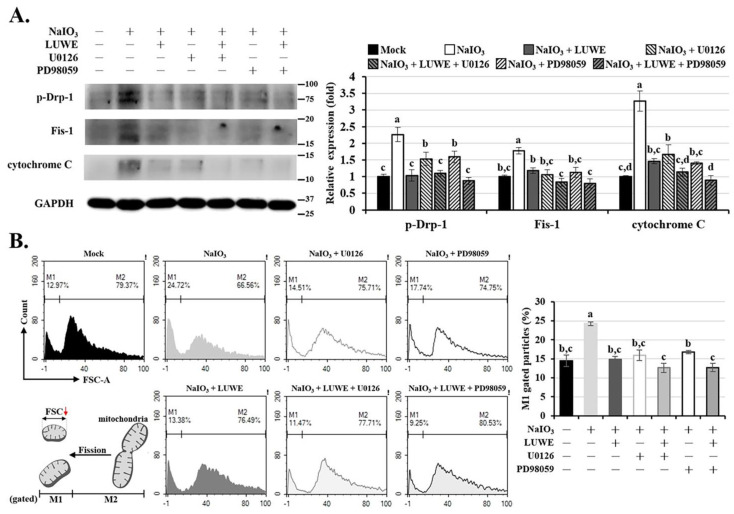
Effects of LUWE, U0126, and PD98059 on NaIO_3_-induced mitochondrial homeostasis. ARPE-19 cells were pretreated with 10 µM U0126 or 20 µM PD98059 mixed with 2.5 mg/mL LUWE or not for 1.5 h, and then cotreated with sodium iodate for 24 h. (**A**) Cell lysates were collected for analysis of p-Drp-1, Fis1, and cytochrome c protein expression by Western blotting. Protein expression was represented by the fold of the mock group. (**B**) The isolated mitochondria from cells in each group were analyzed for particle size using forward scatter (FSC-A) with flow cytometry. All data are expressed as the mean ± SD (*n* = 3). Different letters (a–d) superscripted in the statistical chart indicate that there is statistical significance between groups (*p* < 0.05); on the contrary, being marked with the same letter indicates that there is no statistical significance.

Reportedly, NaIO_3_ can regulate mitochondrial dynamics through ERK and AMPK signaling pathways, involving mitochondrial fission and fusion [12,49]. This process may disrupt mitochondrial membrane potential and promote the release of cytochrome c, triggering a caspase-mediated intrinsic apoptosis cascade [53,54]. Therefore, we conducted protein analysis to determine the expression of factors regulating mitochondrial dynamics. As shown in Figure 6A, NaIO_3_ increased the expression of the mitochondrial division proteins p-Drp-1 and Fis1 in ARPE-19 cells, as well as the expression of cytochrome c; however, LUWE, U0126, or PD98059 can suppress the expression of the aforementioned proteins.

To further determine the influence of LUWE on mitochondrial homeostasis, we isolated mitochondria from cells in each group and measured their size using forward scatter (FSC) in flow cytometry. The results indicate that NaIO_3_ increased the small-sized mitochondrial content (M1-gated particles) in ARPE-19 cells. Meanwhile, LUWE, U0126, or PD98059 can significantly reduce the number of small-sized mitochondria.

Based on the above results, we conclude that LUWE can suppress the mitochondrial oxidative stress-mediated MEK/ERK signaling pathway, thus protecting ARPE-19 cells against mitochondrial fission-stimulated intrinsic apoptosis induced by NaIO_3_.

## 4. Discussion

RPE cell dysfunction is considered a major factor in the onset of AMD. Oxidative stress mediates various physiological mechanisms of RPE cells, including aging, cellular autophagy, and apoptosis [20,31], making antioxidant strategies play a crucial role in slowing the progression of AMD [48].

Citrus fruits are among the most popular fruits globally. Unripe lemons are favored for their acidic juice, but they also generate acidic agricultural waste that cannot be easily disposed of, resulting in additional costs for both the economy and the environment [55]. Reportedly, the polyphenol content in citrus fruits reaches its peak before reaching maturity. This suggests that these by-products of lemons are suitable for extracting antioxidant compounds [56]. In this study, we selected the Eureka variety of lemons referred to in the work of Perez-Perez et al., which is known to have higher contents of flavonoids and polyphenols, commonly found in Southeast Asian regions [57]. Considering the most direct method for handling agricultural waste, we employed water extraction with fresh by-products from lemons, and ultrasound application was applied to shorten the extraction time and enhance the release of antioxidant compounds [26].

It was reported that lemon peel contains 16.71 ± 0.40 mg GAE of polyphenol content and 21.38 ± 1.53 mg TE of ABTS clearance activity per gram (dry weight), extracted using a solvent composed of 50% alcohol and 50% water [58]. Our samples exhibited a polyphenol content of 10.24 ± 0.09 mmole GAE and an ABTS clearance activity of 5.65 ± 0.06 mmole TE (Table 1). Considering that the fresh by-products of lemon contain over 80% water and that most flavonoids and polyphenolic compounds are hydrophobic [59], the above results align with our expectations. In addition, we identified the main components of LUWE as citric acid, limocitrin derivative, neoeriocitrin, p-coumaric acid, and luteolin (Figure 1 and Table 2), which roughly align with those reported by Ledesma-Escobar [43]. Among these, p-coumaric acid has been previously shown to protect lens epithelial cells from MAPK-mediated apoptosis [60], while luteolin can mitigate the inflammatory response regulated by the MAPK pathway in RPE cells [61]. These findings supported our discoveries and suggested the potential of LUWE as a natural compound for retinal injury.

The exact way NaIO_3_ damages RPE cells is not fully known yet. NaIO_3_ triggers oxidative stress and boosts genes linked to dealing with oxidative stress. Mouse models induced by NaIO_3_ mimic some aspects of dry AMD, showing partial loss of RPE cells in patches and the subsequent death of photoreceptor cells and choriocapillaris. Targeting oxidative stress could help ease RPE cell issues and prevent cell death. As shown in Figure 2A, we found that NaIO_3_ caused the destruction and loss of photoreceptor cells in the mouse retina, accompanied by cellular infiltration in the photoreceptor outer segment (OS) and inner segment (IS) regions, a result similar to that of Chowers et al. [62]. At the same time, we also observed that LUWE effectively restricted the development of retinal folds and maintained the structural integrity of each layer. Furthermore, LUWE protected the retina against thickness reduction and cell loss induced by NaIO_3_, primarily occurring in the ONL and INL (Figure 2B–D). These two layers consist of the nuclei of photoreceptor cells and neuronal cells. Their distortion and cell loss directly lead to decreased photosensitivity and visual signal transmission efficiency, making them important indicators for evaluating visual function [63,64]. Thus, the above results indicate that LUWE can mitigate NaIO_3_-induced retinal degeneration.

A previous study indicates that NaIO_3_, by targeting RPE apoptosis, leads to retinal degeneration [12,20,56,65]. In our study, we observed through immunofluorescence staining that LUWE can significantly suppress the expression of cleaved caspase-3 induced by NaIO_3_. This protein was specifically expressed in the RPE cells at the base of the retina, while there was no expression in other locations (Figure 2E). This phenomenon may be due to severe RPE damage induced by NIO_3_ in mice, followed by visual impairment, functional impairment, and photoreceptor loss. The above results suggest that LUWE may protect the retina against NaIO_3_-induced degeneration by preventing RPE cell apoptosis.

To understand the protective mechanisms, we observed the protection of LUWE using NaIO_3_-induced ARPE-19 cells based on previous studies [20,31,66]. We observed that 2.5 mg/mL LUWE can effectively suppress NaIO_3_-induced ARPE-19 cell death (Figure 3B). Therefore, we used annexin V/PI staining to determine the protective pathway of LUWE against cell death, which method has been widely used to distinguish between necrotic and apoptotic cells [67]. The results demonstrate that LUWE effectively reduces the number of apoptotic cells induced by NaIO_3_ (Figure 3D), characterized by a swollen and vacuolated morphology (Figure 3C). Based on the above, we further analyzed the protein expression of apoptotic proteins. The results showed that LUWE effectively suppressed the protein expression of both full-length and activated forms of caspase-3 and PARP induced by NaIO_3_. These findings confirm the protective effect of LUWE against NaIO_3_-induced apoptosis in RPE cells, supporting our observations in the mouse retina.

The MAPK signaling pathway is well known as a central regulator, mediating the cellular response of RPE cells to oxidative stress [12,49]. We used MitoSOX Red staining to confirm that LUWE effectively reduces the mitochondrial ROS levels induced by NaIO_3_ (Figure 4A), thereby inhibiting MAPK signals, especially the MEK/ERK pathway (Figure 4B). The sequential relationship between them has been established in previous literature [4,68]. To observe the importance of the MEK/ERK pathway in the protection of RPE cell damage by LUWE, we pretreated ARPE-19 cells with PD98059 and U0126 before NaIO_3_ treatment, which have been previously confirmed to inhibit ERK signaling through different pathways [51]. Through the CCK-8 assay and annexin V/PI staining, we observed that U0126 and PD98059 can significantly reduce the number of apoptotic cells induced by NaIO_3_ (Figure 5A,B). Furthermore, we observed a significant decrease in the protein expression of cleaved caspase-9 and cleaved PARP induced by NaIO_3_ in the pretreatment groups with LUWE, U0126, and PD98059 (Figure 5C). Therefore, our data reveal that LUWE can suppress the NaIO_3_-induced MEK/ERK pathway, thereby blocking an intrinsic apoptotic pathway.

Past studies have confirmed that NaIO_3_ can disrupt mitochondrial dynamics and activity through the ERK signaling pathway [49,69]. However, further exploration is needed to determine whether targeting ERK-mediated mitochondrial homeostasis can be a viable strategy for formulating AMD treatment approaches. Here, we observed that LUWE can suppress the expression of pro-fission proteins p-Drp-1 and Fis1 induced by NaIO_3_, thereby stabilizing the mitochondrial dynamics and reducing the expression of cytochrome c in ARPE-19 cells (Figure 6A). It is worth noting that the aforementioned mitochondrial damage was also decreased to varying degrees with the pretreatment of U0126 and PD98059. These results demonstrate that LUWE can inhibit mitochondrial ROS-mediated fission, thereby alleviating the intrinsic apoptosis of RPE cells and protecting against retinal degeneration. As reported by Chan et al., our data also indicate that the mitochondrial damage induced by NaIO_3_ is regulated through the MEK/ERK pathway [49].

## 5. Conclusions

**Figure 7:**
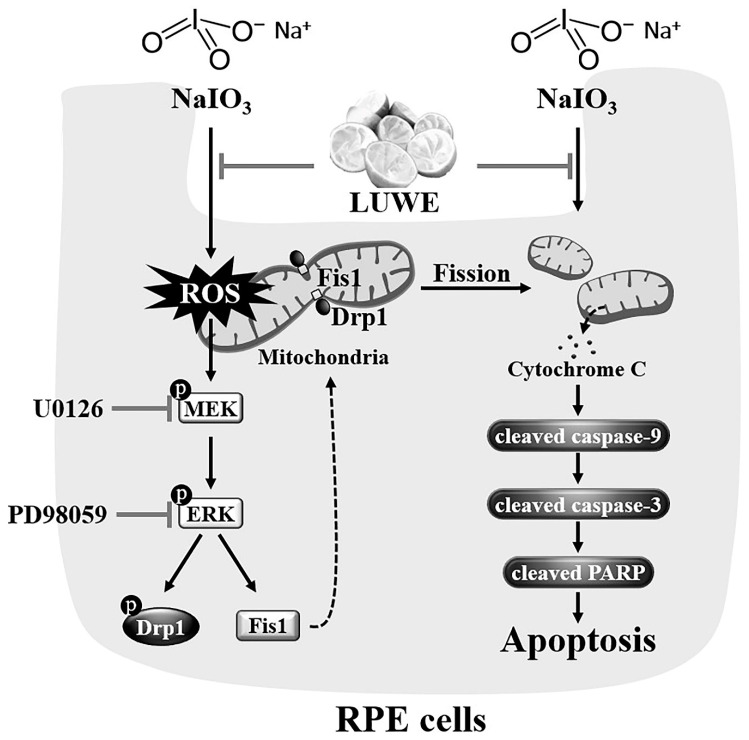
Protection of LUWE against sodium iodate-induced apoptosis in RPE cells. LUWE mitigates mitochondrial ROS-mediated MEK/ERK signaling, consequently reducing mitochondrial fission regulated by Drp-1 and Fis1. As a result, LUWE suppresses the expression of cytochrome c protein, leading to a reduction in RPE cell apoptosis regulated through the caspase-9/caspase-3/PARP cascade. LUWE protected against sodium iodate-induced RPE cell death, thereby reducing retinal thinning and distortion.

This study establishes the effectiveness of isolating antioxidant components from lemon by-products. Using in vitro and in vivo models of NaIO_3_-induced retinal degeneration, we demonstrated that lemon by-product extracts can inhibit ROS-mediated MEK/ERK signaling, thereby preventing mitochondrial fission-triggered RPE cell apoptosis and subsequent neuroretina and photoreceptor loss (Figure 7). Our study not only highlights the innovation of using lemon by-product water extracts for maintaining ocular health but also provides insights and development strategies for functional materials targeting physiological barrier organs like the brain or eyes.

## Figures and Tables

**Table 1:** Antioxidant activity of LUWE.

	Content
ABTS+ scavenging activity (mmole TE/g sample)	5.65 ± 0.06
DPPH scavenging activity (mmole CE/g sample)	2.51 ± 0.03
Polyphenol content (mmole GAE/g sample)	10.24 ± 0.09
Flavonoids content (µmole CE/g sample)	964 ± 46.61

The data are given as the mean ± SEM (*n* = 3). TE, Trolox equivalent; CE, catechin equivalent; GAE, gallic acid equivalent.

**Table 2:** Identification parameters for the 24 flavonoids tentatively identified include the flavonoid name, neutral mass, retention time (R.T.), adduct formed, and main product ions (*m*/*z*).

No.	Compound	Neutral Mass	R.T. (min)	Adduct	Main *m*/*z* Fragments	References
1	Isocitric acid	192.12	2.95	[M+H]^−^	111.00, 390.90	[35,36]
2	Citric acid	192.12	3.83	[M+H]^−^	111.00, 390.90	[36,37,38]
3	Gentiopicroside	356.10	6.53	[M+H]^−^	91.00, 191.00, 719.05	[37]
4	Ferulic acid hexoside	356.32	7.57	[M+H]^−^	91.00, 191.00, 615.15	[38]
5	Quercetin-3-O-rutinoside-7-O-glucoside	772.66	10.12	[M+H]^−^	365.05	[39]
6	Quinic acid	192.06	10.55	[M+H]^+^	83.05, 354.95, 523.05	[35]
7	Limocitrol-O-Glu	538.46	14.72	[M+H]^+^	83.05	[38]
8	Chrysoeriol-6,8-di-C-Glu/stellarin-2	624.50	15.20	[M+H]^−^	91.00, 757.10	[38,40,41,42]
9	p-Coumaroyl quinic acid	338.10	15.73	[M+H]^−^	391.05, 577.05, 755.10	[36]
10	Apigenin-7-O-Neo/rhoifolin	578.16	15.73	[M+H]^−^	336.95, 391.05, 755.10	[38,40,42]
11	Limocitrin-Glu-HMG-Glu	816.22	18.10	[M+H]^−^	391.05	[35,38,43]
12	Sinapoyl-O-glucoside	386.35	20.23	[M+H]^−^	91.00, 336.90	[36,38]
13	Eriodictyol-7-O-Rut/neoeriocitrin	596.17	21.67	[M+H]^−^	659.15	[35,43]
14	Naringenin-7-O-Neo/naringin	580.18	26.30	[M+H]^−^	91.00, 501.00	[35,39,41,43]
15	Hesperetin	302.08	29.17	[M+H]^−^	471.05, 681.15	[35,36,39,43]
16	Hesperetin-7-O-Rut/hesperidin	610.19	29.32	[M+H]^−^	300.95, 471.05	[35,39,41,43,44]
17	Hesperetin-7-O-Neo/neohesperidin	610.19	29.32	[M+H]^−^	301.10, 471.05	[35,36,43]
18	Limonexic acid/limonexin	502.52	30.98	[M+H]^+^	83.05, 687.10	[36]
19	Limocitrol-O-Glu-HMG	682.17	31.38	[M+H]^−^	579.05	[35,36,43]
20	Naringenin-7-O-Neo/naringin	580.18	32.10	[M+H]^−^	91.05, 409.10, 543.10	[35,39,41,43]
21	p-Coumatric acid	164.05	32.95	[M+H]^−^	119.05, 350.95	[36]
22	Luteolin	286.24	34.52	[M+H]^+^	83.05	[36,39]
23	Limocitrin	346.29	34.92	[M+H]^+^	83.05, 797.10	[36]
24	Limocitrol	376.31	39.40	[M+H]^−^	91.05, 435.05, 841.15,	[36]

Glu, glucoside; Neo, neohesperidoside; Rut, rutinoside; HMG, 3-hydroxy-3-methyl-glutaryl.

## Data Availability

Data is contained within the article.
